# VKORC1-dependent pharmacokinetics of intravenous and oral phylloquinone (vitamin K1) mixed micelles formulation

**DOI:** 10.1007/s00228-012-1362-y

**Published:** 2012-08-05

**Authors:** Milka Marinova, Dieter Lütjohann, Olof Breuer, Heike Kölsch, Philipp Westhofen, Matthias Watzka, Martin Mengel, Birgit Stoffel-Wagner, Gunther Hartmann, Christoph Coch, Johannes Oldenburg

**Affiliations:** 1 Institute of Experimental Hematology and Transfusion Medicine, University Clinics Bonn, Sigmund- Freud-Strasse 25, 53127 Bonn, Germany; 2 Institute for Clinical Chemistry and Clinical Pharmacology, University Clinics Bonn, Sigmund- Freud-Strasse 25, 53127 Bonn, Germany; 3 Department of Psychiatry, University Clinics Bonn, Bonn, Germany

**Keywords:** Chromatography, Clinical study, Coagulation, Pharmacokinetics, Phylloquinone, Bioavailability, Clearance

## Abstract

**Objective:**

The pharmacokinetics of phylloquinone (vitamin K1) were evaluated in healthy human adult volunteers (15 male and 15 female) following oral and intravenous administration of a mixed micelles formulation (Konakion® MM 2 mg) in an open label study design. The subjects were allocated to one of three genotype-specific groups (*n* = 10 in each group) in terms of *VKORC1* promoter polymorphism c.-1639 G > A to explore the relationship between genotype and pharmacokinetic parameters.

**Methods:**

Blood samples were collected for up to 24 h after administration. Phylloquinone serum levels were determined by reversed phase HPLC with fluorometric detection after post-column zinc reduction. Pharmacokinetic evaluation was performed using non-compartmental analysis.

**Results:**

Pharmacokinetic analysis of serum phylloquinone concentration versus time profiles revealed significant differences in the main pharmacokinetic parameters between groups. Upon oral administration, *VKORC1* AG carriers showed 41 % higher mean bioavailability (*p* = 0.01) compared with homozygous AA individuals. Furthermore, AG subjects exhibited 30 % (*p* = 0.042) and 36 % (*p* = 0.021) higher mean AUC compared with GG and AA respectively. Terminal half-life was 32 % and 27 % longer for AG carriers in comparison to GG (*p* = 0.004) and AA (*p* = 0.015) genotypes respectively.

**Conclusion:**

Pharmacokinetic differences indicated significant inter-individual variance of vitamin K fate in the human body. The influence of the *VKORC1* promoter polymorphism c.-1639 G > A on the pharmacokinetic properties of phylloquinone could be demonstrated in humans. To gain deeper insight in other potential genetic determinants of systemic vitamin K exposure, further correlation of the phenotype–genotype relationship of different players in vitamin K turnover has to be gained.

## Introduction

Vitamin K is a fat-soluble vitamin needed for a unique post-translational chemical modification of proteins with calcium-binding properties, collectively known as vitamin K-dependent proteins (VKDPs) or Gla-proteins. Vitamin K is mostly required for blood coagulation, but it is also involved in metabolic pathways in bone and other tissue. It acts as a cofactor in the synthesis of active blood-clotting factors II, VII, IX, X, protein C and protein S as well as non-coagulation proteins such as osteocalcin and matrix Gla protein [1, 2]. Activation of VKDPs is linked to an enzymatic cycle, denoted the vitamin K cycle, which carries out both γ-glutamyl carboxylation of vitamin K and serves as a salvage pathway to recover vitamin K from its epoxide metabolite for reuse in carboxylation [3]. The latter reaction is catalyzed by Vitamin K epoxide reductase (VKOR). In 2004, the gene encoding vitamin K epoxide reductase complex subunit 1 (VKORC1), the key protein in the vitamin K cycle, was identified and characterized [4, 5]. The activity of the VKOR can be blocked by warfarin and other coumarin-based drugs [6, 7]. Common polymorphisms such as *VKORC1* promoter polymorphism c.-1639 G > A and haplotypes within the *VKORC1* gene have been associated with interindividual variability in the warfarin dose required for therapeutic anticoagulation and show increased coumarin sensitivity [8–12].

The major dietary form of vitamin K is phylloquinone (vitamin K1), which is produced by green plants and present in foods of plant origin, especially in green leaf and flower vegetables [13]. Vitamin K is normally absorbed with other fat-soluble vitamins predominantly from the proximal intestine [14]. This intestinal absorption involves the solubilization of vitamin K into mixed micelles composed of bile salts and products of pancreatic lipolysis and is known to be impaired in patients with malabsorption or other gastrointestinal disorders, including biliary atresia, cystic fibrosis, celiac disease, and short bowel syndrome [15]. Vitamin K is not known to have a carrier protein; instead, triglyceride-rich lipoproteins (TRL), primarily chylomicron remnants and very low-density lipoproteins (VLDL), are thought to be the main transporters of phylloquinone [16–18]. Vitamin K is extensively metabolized in the liver and excreted in the urine (20 %) and bile (40 %).

There has been a recent interest in the role of genetics as a determinant of the interindividual variation in vitamin K status. Nongenetic determinants account for approximately 20 % of the interindividual variation in vitamin K status in Caucasian adults [19]. Potential genetic determinants of vitamin K status include variation in the genes involved in transport or uptake of vitamin K into the tissue, thus influencing tissue-specific availability. Moreover, vitamin K recycling in the liver may be affected by polymorphisms such as that of the aforementioned *VKORC1* gene [8–10]. Further, polymorphisms in cytochrome P450 4 F2 (*CYP4F2*) and ATP-binding cassette sub-family C member 6 (*ABCC6*) genes have been linked to altered vitamin K metabolism [20–22]. For instance, the presence of V433M polymorphism (rs2108622) in *CYP4F2* (haplotype CYP4F2*3) caused elevated hepatic vitamin K1 levels, necessitating a higher warfarin dose to reach therapeutic anticoagulation response [20].

Examining different phenotype–genotype relationships will elucidate the potential genetic determinants of the vitamin K status. In this regard, the phenotype refers to physiological processes such as absorption, distribution, metabolism, and elimination of vitamin K. Therefore, the primary aim of this phase I clinical study was to explore the possible effect of the *VKORC1* promoter polymorphism c.−1639 G > A on the pharmacokinetics of vitamin K1 in humans.

## Materials and methods

### Subjects and study design

The study was registered in the European Clinical Trials Database (EudraCT number 2008-003643-36) and conducted in accordance with Good Clinical Practice (GCP), the current requirements of EMA (European Medicines Agency) [23], the Declaration of Helsinki and local and European law. The phase I study protocol was approved by the Ethics Committee responsible (the Medical Faculty of the Rheinische Friedrich-Wilhelms University Bonn) and by the Federal Institute for Drugs and Medical Devices in Germany (BfArM). Each volunteer participating in this study was informed by the clinical investigator about the modality and the possible risks of the trial and consented to study participation in writing before undergoing the first study-related procedure. Prior to and after the study treatment, volunteers underwent safety clinical and laboratory screening. Excluded from the study were: women of child-bearing potential without reliable contraception, pregnant or lactating women, persons with any disease likely to disturb the vitamin K metabolism such as bleeding or thromboembolic history (acute or persistent) or dysfunctions of intestinal lipid absorption (Crohn’s disease, cholestatic liver diseases).

Fifteen men (28 ± 6 years, BMI 24 ± 2 kg/ m^2^) and 15 women (29 ± 7 years, BMI 21 ± 2 kg/ m^2^) of Caucasian origin, aged 22–46 years, participated in this study. Of these, 5 women and 5 men belonged to each VKORC1 genotype-specific group [9] as described below. All subjects were healthy according to their medical history, physical examination, and standard laboratory procedures. The subjects refrained from any other medication from 10 days prior to enrolment until the end of the study. Vitamin K1 was administered as an investigational product in its synthesized form *phytomenadione*, which is available as the liquid formulation Konakion® MM containing 2 mg/200 μL in a mixed micelles (MM) solution [24, 25]. Konakion® MM 2 mg is approved for prophylaxis and therapy of vitamin K deficiency bleeding in newborns. In this clinical trial, vitamin K1 pharmacokinetics was investigated in healthy volunteers after oral and intravenous route of administration. All participants were in a fasting state when Konakion® MM was administered. Standardized food was served no earlier than 10 h after dosing.

A single oral dose of 2 mg Konakion® MM was followed by a wash-out period of 7 days and a single intravenous administration of the same dosage using an open label study design. Pharmacokinetic parameters of the two routes of administration were calculated and compared. The i.v. injection was carried out into a vein of one forearm, opposite to the one from which blood samples were taken. Systemic and local tolerance of the investigational product were carefully checked by the clinical investigator. Blood samples (7.5 mL each) were collected into serum gel tubes up to 24 h following p.o. and i.v. vitamin K1 administration: at 1, 2, 2.5, 3, 3.5, 4, 4.5, 5, 5.5, 6, 6.5, 7, 7.5, 8, 9, 10, 24 h after p.o. dosing, and at 2, 5, 10, 20, 30, 40, 50 min, and 1, 1.5, 2, 2.5, 3, 3.5, 4, 4.5, 5, 5.5, 6, 6.5, 7, 8, 9, 10, and 24 h after i.v. injection. During the first 10 h, blood was collected through a peripheral vein catheter. Serum was separated immediately by centrifugation at 2,250 *g* for 10 min at 18°C and stored protected from light at −20°C prior to analysis.

### Genotyping

Confirmatory genotyping was performed to ensure that the study subjects were equally distributed among the three genotype groups of interest regarding *VKORC1*: c.−1639 G > A promoter polymorphism. Genotyping for the SNP rs9923231 in the *VKORC1* gene was performed using a TaqMan® Allelic Discrimination Assay based on fluorescence-labeled probes (primer and probe details as described previously [26]). Subjects who were homozygous GG carriers, heterozygous AG carriers as well as homozygous AA genotypes were included (each group consisted of 5 men and 5 women). Within the Caucasian population, the allele frequency is 43 % for the A allele and 57 % for the G allele [9]. Furthermore, confirmatory genotyping was performed retrospectively with regard to SNPs in the *CYP4F2* (V433M polymorphism, C > T [20]) and *ABCC6* (promoter polymorphism c. −127 C > T [21, 22, 27]) genes involved in vitamin K metabolism and elimination.

### Analysis of vitamin K1 in serum

Vitamin K1 was determined by reversed phase HPLC combined with fluorometric detection following post-column zinc reduction as described previously [28]. Briefly, the vitamin K related compounds were purified from serum by liquid–liquid extraction using *n*-hexane. After the addition of the appropriate amount of internal standard (0.5, 1 or 2 ng per absolute injection), the upper hexane layer was quantitatively collected, pooled, and evaporated to dryness under a gentle stream of nitrogen at 50°C. Vitamin K isolation from lipid extracts was performed by using a solid phase extraction (SPE) system. The separated and dried vitamin K fraction was dissolved in 50 μL of 2-propanol and subjected to HPLC analysis. Vitamin K compounds, all in the nonfluorescent quinone forms, were separated by an isocratic HPLC system on a reversed phase-C18 column and then converted to their fluorescent hydroquinone forms by post-column zinc reduction. The mobile phase contained, per liter, 880 mL of methanol, 100 mL of acetonitrile, 1.1 g of zinc acetate, 10 mL of acetic acid, and 10 mL of water. The flow rate was 0.8 mL/ min and the detection wavelengths were 246 nm excitation, 430 nm emission. The limits of detection and quantification were 0.015 ng mL^−1^ and 0.15 ng mL^−1^ respectively. High sensitivity, analytical recoveries, accuracy, and calibration curve linearities could be reached, as shown before [28]. The within-day and day-to-day coefficients of variation amounted to less than 10 %, while the recovery ranged from 91 % to 114 %. The accuracy was proven by good results from external quality assurance [29]. Long-term stability was verified over a period of 6 months.

### Instrumentation, reagents, disposables, and standards

The isocratic HPLC system (VWR, Darmstadt, Germany) consisted of separation Hitachi modules with an in-line vacuum degasser, an L-2130 pump, an L-2200 autosampler, an L-2300 column oven, and an L-2485 fluorescence detector, all controlled by Elite LaChrom software (V.3.1.7.). A Nucleodur C18 Gravity column (3 mm i.d. × 100 mm in length, 3-μm particle size) was used for separation. The lipid compounds were separated using a solid-phase extraction vacuum manifold. The analytical reagent grade chemical zinc acetate (99.99 % metallic basis) was obtained from Sigma–Aldrich (Hamburg, Germany). Acetic acid for vitamin K determination assay was purchased from VWR International (Darmstadt, Germany). All solvents used were HPLC grade (LiChrosolv®) and obtained from VWR (Darmstadt, Germany). Disposable SPE Cartridges Chromabond®SiOH 3 mL/ 500 mg were supplied by Macherey-Nagel (Düren, Germany). Vitamin K, i.e., phylloquinone and menaquinone-4, with a purity of 99 % were purchased from Sigma–Aldrich (Hamburg, Germany). The internal standard (ISTD) vitamin K1(25) [30] as a 10-mg/mL solution was kindly provided by Dr. Schurgers, University of Maastricht, the Netherlands. Konakion® MM 2 mg was purchased from Roche (Grenzach-Wyhlen, Germany).

### Pharmacokinetic and statistical analysis

Pharmacokinetic parameters were determined for each subject using noncompartmental analysis with WinNonlin (version 5.2.1., Pharsight Corp., CA, USA). Serum concentration – time data of vitamin K1 was considered the primary variable as well as the evaluation of pharmacokinetic parameters including *maximum serum concentration* (C_max_), *time of the maximum serum concentration* (t_max_), *area under the curve from time zero to infinity* (AUC), *bioavailability* (F_p.o._), *clearance* (CL), *volume of* *distribution at steady state* (V_ss_), *terminal half-life* (t_½_).

AUC was calculated by AUC = AUC_0-t_ + C_last_ / λ_z_ using the linear–log trapezoidal method. The terminal (first-order) elimination rate constant (λ_z_) was calculated from the slope of the terminal linear portion of the log concentration vs time curve by linear regression analysis. The mean residence time (MRT) was calculated as AUMC/AUC where AUMC is the area under the first moment curve. V_ss_ was calculated as: V_ss_ = MRT × CL. The F_p.o._ of Konakion® MM was calculated as: F_p.o._ = AUC _p.o._ / AUC _i.v._ × Dose _i.v._ / Dose _p.o._ x 100 % . The systemic CL was calculated according to the equation: CL = Dose _i.v._ / AUC _i.v._.

t_½_ was calculated using the terminal elimination rate constant as: t_½_ = 0.693/ λ_z_.

The plasma concentration values were corrected for endogenous vitamin K levels in each subject by the subtraction of individual pre-dose values from all post-dose values. The plasma concentration was set to zero in instances where such correction resulted in small negative values.

Linear regression, mean, median, standard deviation (SD), and standard error of the mean (SEM) were calculated using Microsoft Excel. Statistical evaluation was performed using SPSS (IBM SPSS Statistics 19.0). Univariate analyses of variance (*uANOVA*) were performed including gender as a factor and Tukey post-hoc testing. Thus, significance differences in various pharmacokinetic parameters, such as F_p.o._, t_½_, AUC etc., could be proven.

## Results

**Fig. 1:**
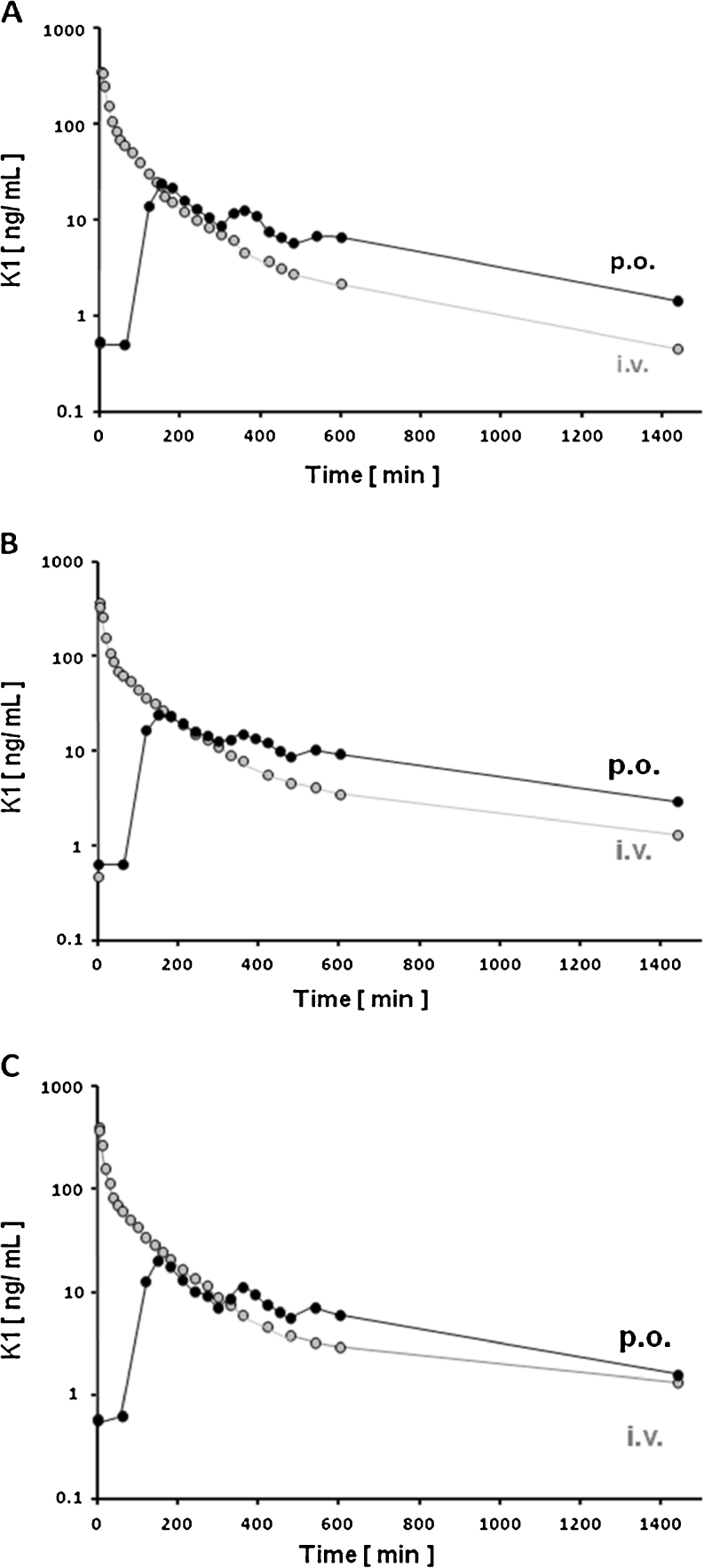
Serum concentration (log scale) vs time profiles of vitamin K1 for each *VKORC1* group (*VKORC1* promoter polymorphism c. −1639 G > A) following p.o. and i.v. administration. **a** Group GG, **b** Group AG, **c** Group AA

Thirty healthy volunteers were included in the clinical study on the pharmacokinetics of vitamin K1. Baseline characteristics were balanced across *VKORC1* genotype groups. The plasma concentration–time profiles of vitamin K1 were obtained by HPLC-RP18 with fluorescence detection. All evaluated pharmacokinetic parameters obtained by noncompartmental analysis were normally distributed. Pharmacokinetic data for each genotype GG, AG, and AA *VKORC1*:c.-1639 G > A are summarized in Table 1. Some of the main parameters, such as C_max_ and t_max_, were estimated directly from the data. Figure 1 represents mean vitamin K1 serum concentrations (*n* = 10 for each time point), expressed on a logarithmic scale, vs time profiles, obtained after p.o. intake of 2 mg Konakion® MM and following i.v. administration of the same dosage for each *VKORC1* group (Fig. 1a regarding GG, Fig. 1b AG, and Fig. 1c AA). Phylloquinone concentration–time curves varied between the volunteers as well as when p.o. and i.v. experiments were compared, although the general curve shape following each route of administration remained similar. The i.v. plasma concentration–time curves showed a biexponential decline, suggesting a short initial distribution phase followed by an elimination phase. The curves obtained after the p.o. route of administration showed an initial absorption phase with a roughly monoexponential decay after C_max_ was reached. However, there was also a second and a third distinct maximum following C_max_, which is typical for substances undergoing enterohepatic circulation.

**Table 1:** Differences in vitamin K1 pharmacokinetic parameters between *VKORC1*: c.−1639 G > A groups (*uANOVA* with regard to gender)

Pharmacokinetics parameter	Genotype *VKORC1*: c.−1639 G > A			*P* value *uANOVA*
	GG	AG	AA	
p.o.				
t_max_ (min)	181 ± 67^b^	210 ± 119	217 ± 132	n.s.
	(120–362)	(119–481)	(122–542)	
C_max_ (ng mL^-1^)	29 ± 11	30 ± 10	27 ± 12	n.s.
	(15–55)	(14–44)	(6–46)	
AUC (ng h mL^-1^)	145 ± 68	206 ± 79*^, §^	132 ± 50	0.015
	(94–278)	(92–364)	(45–211)	
F_p.o._ (%)	51 ± 20	73 ± 33^§^	43 ± 13	0.011
	(23–90)	(38–150)	(27–70)	
CL^a^ (mL min^-1^)	253 ± 80	165 ± 76	286 ± 160	n.s.
	(116–331)	(72–338)	(154–679)	
t_½_ (min)	311 ± 54	457 ± 141^$, §^	332 ± 48	0.003
	(250–394)	(313–787)	(261–429)	
i.v.				
AUC (ng h mL^-1^)	298 ± 97	318 ± 48	319 ± 78	n.s.
	(204–510)	(238–390)	(177–453)	
V_ss_ (L)	23 ± 8	25 ± 13	19 ± 5	n.s.
	(11–35)	(10–58)	(12–27)	
CL (mL min^-1^)	118 ± 32	104 ± 19	109 ± 33	n.s.
	(64–164)	(70–136)	(71–187)	
t_½_ (min)	436 ± 186	492 ± 385	383 ± 114	n.s.
	(133–727)	(194–1543)	(158–548)	

*p < 0.05; significantly different from GG (Post-hoc with Tukey)

^§^ *p* < 0.05; significantly different from AA (Post-hoc with Tukey)

^$^ *p* < 0.01; significantly different from GG (Post-hoc with Tukey)

^a^Clearance following oral (p.o.) administration CL/F

^b^Mean values ± standard deviation with range in parenthesis; *n* = 10 in each group

**Fig. 2:**
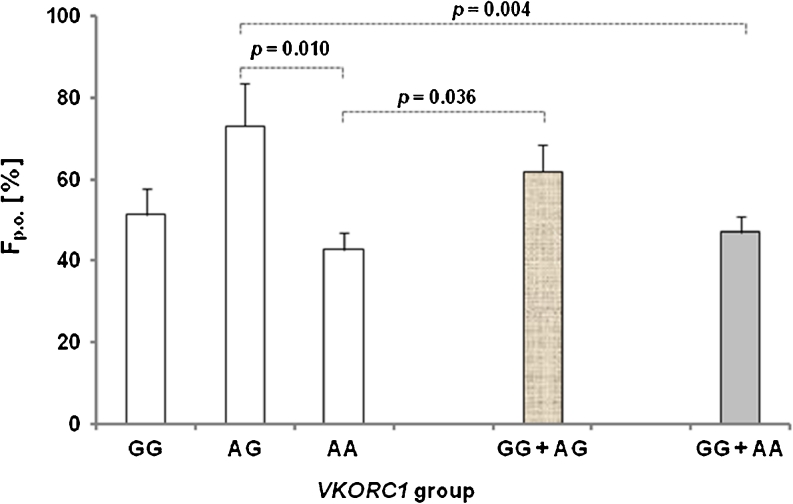
Significant differences (*uANOVA*) in bioavailability among *VKORC1* groups (AG to AA; AG to GG + AA; AA to GG + AG) following vitamin K1 p.o. administration

Following oral vitamin K1 administration, pharmacokinetic parameters such as AUC, F_p.o._, and t_½_ showed statistically significant differences between the *VKORC1* genotype groups (*uANOVA* model; corresponding *p* values are listed in Table 1). AG subjects exhibited 30 % (*p* = 0.042) and 36 % (*p* = 0.021) higher mean AUC compared with GG and AA individuals respectively. The mean bioavailability for AG carriers was 41 % higher (*p* = 0.01; Fig. 2) than for homozygous AA individuals. Statistically significant differences in terminal half-life between groups were also observed. AG genotype carriers showed 32 % and 27 % longer t_½_ to GG (*p* = 0.004) and AA (*p* = 0.015) genotype carriers respectively. Furthermore, the bioavailability was on average significantly higher for men than for women (66 % vs 45 % respectively; *p* = 0.024).

**Fig. 3:**
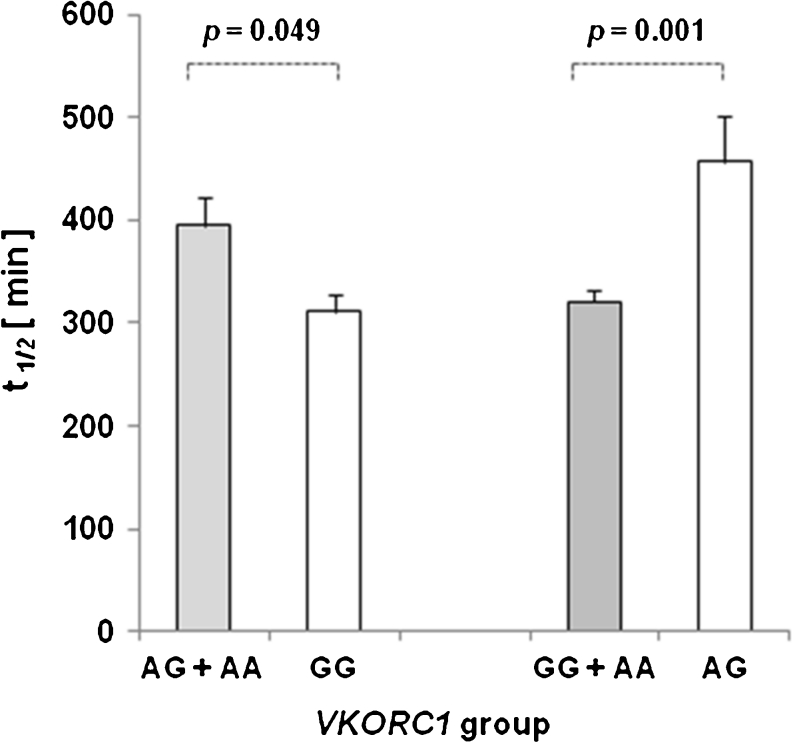
Significant differences (*uANOVA*) in elimination half-time among *VKORC1* groups (AG + AA to GG; GG + AA to AG)

Interestingly, statistically significant differences in bioavailability (Fig. 2) and terminal half-life (Fig. 3) were also found when the results from both groups of homozygous individuals (GG + AA) were pooled and compared with those of heterozygous subjects (AG). In addition, homozygote carriers of the A-allele showed lower F_p.o._ mean values than GG + AG genotype carriers (Fig. 2), whereas homozygous carriers of the G-allele exhibited shorter t_½_ than carriers of at least one A allele (AG + AA) (Fig. 3).

Following intravenous vitamin K1 administration, statistically significant differences between VKORC1 genotype groups were not observed for pharmacokinetic parameters AUC, V_ss_, CL, and t_½_ by application of *uANOVA* analysis (mean values shown in Table 1).

To investigate the role of polymorphisms of other genes such as *CYP4F2* and *ABCC6* on vitamin K pharmacokinetics, differences in main pharmacokinetic parameters were assessed in relation to these genotypes (Table 2). For both *CYP4F2* and *ABCC6*, two groups were selected: one group of homozygous wild-type carriers CC and one group of individuals with at least one T allele (CT + TT). This was due to the very small number of TT carriers (*n* = 1 for *CYP4F2* and *n* = 1 for *ABCC6*). For the V433M polymorphism of the *CYP4F2* gene, mean V_ss_ values differed significantly between the two groups (*uANOVA*; *p* = 0.036) and were lower for the wild-type individuals. Between *ABCC6* genotypes considerable differences in i.v. V_ss_ mean values were also observed (*uANOVA*; *p* = 0.031), in that V_ss_ values were lower for the wild-type group (CC genotype).

**Table 2:** Differences in vitamin K1 pharmacokinetic parameters between *CYP4F2* (V433M polymorphism; haplotype CYP4F2*3) and *ABCC6* (promoter polymorphism c. −127 C > T) group CC and group CT + TT

Pharmacokinetics parameter	*CYP4F2*		*ABCC6*	
	CC	CT + TT	CC	CT + TT
	(*n* = 20)	(*n* = 10)	(*n* = 21)	(*n* = 9)
p.o.				
t_max_ (min)	209 ± 111^b^	190 ± 104	211 ± 120	184 ± 69
	(119–542)	(122–481)	(119–542)	(122–360)
C_max_ (ng mL^-1^)	29 ± 13	29 ± 8	29 ± 13	28 ± 6
	(6–55)	(14–38)	(6–55)	(20–38)
AUC (ng h mL^-1^)	160 ± 82	163 ± 51	154 ± 81	176 ± 49
	(45–364)	(96–262)	(45–364)	(106–262)
F_p.o._ (%)	56 ± 30	54 ± 17	52 ± 28	63 ± 19
	(23–150)	(32–90)	(23–150)	(32–92)
CL^a^ (mL min^-1^)	248 ± 138	208 ± 69	255 ± 133	187 ± 66
	(72–679)	(116–331)	(72–679)	(116–307)
t_½_ (min)	366 ± 126	367 ± 74	349 ± 76	409 ± 164
	(250–787)	(285–524)	(250–592)	(259–787)
i.v.				
AUC (ng h mL^-1^)	310 ± 81	322 ± 63	315 ± 78	303 ± 71
	(177–510)	(219–390)	(177–510)	(204–390)
V_ss_ (L)	20 ± 6*	27 ± 13	20 ± 6*	28 ± 13
	(10–35)	(12–58)	(11–33)	(10–58)
CL (mL min^-1^)	112 ± 30	106 ± 25	110 ± 28	112 ± 31
	(64–187)	(70–148)	(64–187)	(70–164)
t_½_ (min)	386 ± 135	539 ± 383	402 ± 120	519 ± 425
	(133–707)	(158–1,543)	(194–707)	(133–1,543)

^*^ *p* < 0.05; significantly different from CT + TT *ABCC6*; uANOVA (with regard to gender)

^a^Clearance following oral (p.o.) administration CL/F

^b^Mean values ± standard deviation with range in parenthesis

Correlation analysis between vitamin K1 pharmacokinetic parameters and fasting plasma triglycerides was performed without stratification for *VKORC1* genotypes. For each route of phylloquinone administration (p.o. and i.v), triglyceride levels inversely correlated significantly with CL (*p* < 0.05), showing Pearson product–moment correlation coefficients of about −0.4 (data not shown).

## Discussion

The present exploratory phase I clinical study was designed to determine inter-individual pharmacokinetic variance of vitamin K fate in the human body. Furthermore, the possible effect of the *VKORC1* promoter polymorphism c. −1639 G > A on the metabolism of phylloquinone, by means of correlating pharmacokinetic properties with the different genotypes in humans was investigated. There are only a few reports about the pharmacokinetics of vitamin K after oral, intravenous or intramuscular single-dose administration [31–35]. The investigation was performed in a representative group of healthy volunteers. The trial showed a statistically significant difference in bioavailability after oral administration of phylloquinone among *VKORC1* genotype groups (particularly by comparison of AG individuals with AA carriers). F_p.o._ values were in accordance with data from previous studies [28] and revealed substantial inter-individual variability. It is noteworthy that the highest bioavailability was found in heterozygotes, carriers of the AG genotype; furthermore, it was higher for male than for female subjects, although this finding cannot be physiologically and clinically eligible. Vitamin K is mostly required for blood coagulation, but is also involved in metabolic pathways in bone and other tissue. In view of the multifunctional role of this vitamin in the human body, an evolutionary enrichment in individuals in the population with the potential for high vitamin K exposure seems reasonable. However, the mechanism by which the VKORC1 AG genotype yields the highest bioavailability compared with the other polymorphisms remains to be elucidated. Moreover, it must be noted that AA genotype carriers showed the lowest phylloquinone bioavailability. This fact could play a role, for instance, when vitamin K adjustment in unstable patients under warfarin therapy is required, or when vitamin K has to be supplemented orally to antagonize intoxication with rodenticides or is given to over-anticoagulated patients therapeutically. However, the safest approach to giving vitamin K to patients remains high-dose administration without gene dose adjustment.

Considering retrospectively other genes involved in vitamin K metabolism and tissue distribution, such as *CYP4F2* and *ABCC6* polymorphisms, a considerable difference in intravenous V_ss_ between phenotypes was shown, whereas other pharmacokinetic parameters do not seem to be influenced. However, to obtain a deeper insight into such a relationship further investigations with more subjects are needed. Because of the small number of homozygous polymorphism carriers, possible differences among the three groups could not be distinguished, perhaps missing a potential distinction in pharmacokinetics.

Terminal half-lives following oral phylloquinone administration were considerably longer for *VKORC1* heterozygotes than for the homozygous carriers. This observation also remained valid after pooling both homozygous groups, GG and AA genotypes, together and comparing them with the AG genotype carriers. The terminal half-lives showed larger variation following intravenous administration than those following oral dosing. Hence, there was a tendency, albeit not a statistically significant one, toward longer half-lives in VKORC1 heterozygotes also following intravenous administration. The estimated mean terminal half-lives of 6.1 h after oral phylloquinone administration were comparable with previously published data [31]. In contrast, the mean terminal half-life of 7.3 h following intravenous administration was longer than in previous data, but can be explained by one subject showing an extremely long value. It should be noted that all observed significant differences between groups remain valid, even after exclusion of this individual from the analysis.

The plasma concentration–time profiles in most subjects showed at least two additional peaks during the elimination phase following oral dosing. This is sometimes seen with compounds undergoing enterohepatic circulation, but appears unlikely in this case since straight exponential declines were seen after intravenous administration. It is also noteworthy that the absorption was rather slow, so that mean C_max_ was not reached before 3 h. Hence, the most likely explanation for multiple peaks with oral dosing is absorption dependence on food or emptying of bile acids. This is further corroborated by that fact that extra peaks were in synchrony in most subjects, and occurred at time points following each meal intake. Moreover, as a consequence of a protracted absorption phase, plasma concentrations at late time points were higher following oral administration than those after intravenous dosing.

The liver is the primary eliminating organ, as is evident from previously published studies in patients with severe acute liver disease, where phylloquinone serum half-lives were approximately twice as long as those found in subjects with normal hepatic function [34]. Moreover, terminal half-lives of vitamin K1 in newborns were also considerably longer compared with adults [33]. Based on our findings, it can be suggested that the highest phylloquinone bioavailability in *VKORC1* heterozygotes can be aligned with lower clearance and the potential for higher systemic exposure to vitamin K in these subjects. Furthermore, provided that hepatic metabolism is the major route of elimination, lower clearance indicates lower hepatic uptake and/or vitamin K metabolism in VKORC1 AG genotype carriers. Theoretically, differences in body composition (fatty tissue) and transporting triglyceride-enriched lipoproteins (VLDL) [17, 36] could affect tissue distribution and elimination rates for a lipid-soluble compound such as vitamin K. However, in this study there were no significant differences in V_ss_ between groups, and inclusion of plasma triglyceride levels as a potential influencing factor in *uANOVA* did not diminish the significance of differences in F_p.o._ between groups.

In conclusion, significant inter-individual pharmacokinetic variance of vitamin K fate in the human body could be indicated. Further, an influence of the *VKORC1* promoter polymorphism c. −1639 G > A on the pharmacokinetic properties of phylloquinone in humans was shown. Significant differences in main pharmacokinetic parameters, such as bioavailability and terminal half-life between groups, suggest corresponding differences in the processing of vitamin K in the human body. The clinical importance of potential genetic determinants of vitamin K status should be further investigated with respect to effects on absorption, distribution, metabolism, and elimination of vitamin K.
